# Balance between carbon gain and loss under long-term drought: impacts on foliar respiration and photosynthesis in *Quercus ilex* L

**DOI:** 10.1093/jxb/erv492

**Published:** 2015-11-09

**Authors:** D. Sperlich, A. Barbeta, R. Ogaya, S. Sabaté, J. Peñuelas

**Affiliations:** ^1^Departament d’Ecologia, Facultat de Biologia, Universitat de Barcelona, Diagonal 645, 08028 Barcelona, Spain; ^2^CREAF, Cerdanyola del Vallès, 08193 Barcelona, Catalonia, Spain; ^3^CSIC, Global Ecology Unit CREAF-CSIC-UAB, Cerdanyola del Vallès, 08193 Barcelona, Catalonia, Spain

**Keywords:** Carbon-use efficiency, day respiration, *J*_max_, mesophyll conductance, rainfall exclusion, *V*_c,max_.

## Abstract

Terrestrial carbon exchange is a key process of the global carbon cycle consisting of a delicate balance between photosynthetic carbon uptake and respiratory release. We have, however, a limited understanding how long-term decreases in precipitation induced by climate change affect the boundaries and mechanisms of photosynthesis and respiration. We examined the seasonality of photosynthetic and respiratory traits and evaluated the adaptive mechanism of the foliar carbon balance of *Quercus ilex* L. experiencing a long-term rainfall-exclusion experiment. Day respiration (*R*_d_) but not night respiration (*R*_n_) was generally higher in the drought treatment leading to an increased *R*_d_/*R*_n_ ratio. The limitation of mesophyll conductance (*g*_m_) on photosynthesis was generally stronger than stomatal limitation (*g*_s_) in the drought treatment, reflected in a lower *g*_m_/*g*_s_ ratio. The peak photosynthetic activity in the drought treatment occurred in an atypical favourable summer in parallel with lower *R*_d_/*R*_n_ and higher *g*_m_/*g*_s_ ratios. The plant carbon balance was thus strongly improved through: (i) higher photosynthetic rates induced by *g*_m_; and (ii) decreased carbon losses mediated by *R*_d_. Interestingly, photosynthetic potentials (*V*_c,max_, *J*_max_, and *TPU*) were not affected by the drought treatment, suggesting a dampening effect on the biochemical level in the long term. In summary, the trees experiencing a 14-year-long drought treatment adapted through higher plasticity in photosynthetic and respiratory traits, so that eventually the atypical favourable growth period was exploited more efficiently.

## Highlight

We unveil the impacts of a 14-year-long drought treatment on the leaf physiology of *Quercus ilex* showing a higher plasticity in photosynthetic and respiratory traits.

## Introduction

Warmer and drier conditions are expected globally under current climate change scenarios and particularly in the Mediterranean region (Somot *et al.*, 2008; Friend, 2010; IPCC, 2013). Seasonal reoccurring drought is the main natural environmental factor in the Mediterranean region limiting plant growth and yield (Specht, 1969; Di Castri, 1973). Projected water shortages are thus likely to intensify the limitations on plant productivity and forest growth. Several studies have already reported drought-induced forest impacts and diebacks in the Mediterranean region (Peñuelas *et al.*, 2001; Martínez-Vilalta and Piñol, 2002; Raftoyannis *et al.*, 2008; Allen *et al.*, 2010; Carnicer *et al.*, 2011; Matusick *et al.*, 2013), as well as shifts in vegetation composition (Jump and Peñuelas, 2005; Anderegg *et al.*, 2013). Seasonal summer drought limits plant growth and productivity most strongly through reductions in the plant carbon budget, which depends on the balance between photosynthesis and respiration (Flexas *et al.*, 2006). Winter has been somehow overlooked, despite the importance of potential recovery and growth periods for the annual carbon budget, especially for evergreen vegetation (Sperlich *et al.*, 2014, 2015). The Mediterranean region is characterized by a high variability in temperature and precipitation regimes, especially in mountainous areas such as the Prades Mountains in north-eastern Spain (Barbeta *et al.*, 2013). Climate extremes combined with high interannual variability complicate the scaling of carbon dynamics from one year to another (Reynolds *et al.*, 1996; Morales *et al.*, 2005; Gulías *et al.*, 2009). In fact, the modelling performance in Mediterranean-type ecosystems is particularly poor (Morales *et al.*, 2005; Vargas *et al.*, 2013) owing to under-represented soil–water patterns and our limited understanding of the effects of water stress on both carbon uptake and release (Hickler *et al.*, 2009; Niinemets and Keenan, 2014).

The non-photorespiratory carbon release in leaves is called mitochondrial respiration—a central metabolic process that produces energy (ATP, NADPH) and carbon skeletons for cellular maintenance and growth. It also contributes to significant carbon losses—especially under stress conditions—altering the net carbon gain (van Oijen *et al.*, 2010). Although the drought responses of Mediterranean vegetation have been investigated extensively, most studies concern *photosynthetic* responses (reviewed by Flexas *et al.*, 2014), whereas *respiratory* responses in leaves have largely been neglected (Niinemets, 2014). Also, it is not clear how seasonality and other abiotic stressors affect the balance of night respiration (*R*_n_) and day respiration (*R*_d_) in the leaves. This is partly owing to measurement difficulties; *R*_n_ can easily be measured by darkening the leaf, but *R*_d_ is harder to obtain and is traditionally estimated from carbon-response curves with the Laisk method, from light-response curves with the Kok method, or with an amended version of the Kok method with chlorophyll fluorescence developed by Yin *et al.* (2009) (reviewed by Yin *et al.*, 2011). Measurement constraints and lacking research priorities can account for the dearth of data on respiratory responses to abiotic stress, particularly drought (Atkin and Macherel, 2009; Heskel *et al.*, 2014). Wright *et al.* (2006) provided evidence that irradiance, temperature, and precipitation affect respiration in a wide range of woody species around the world; Mediterranean species, however, were not covered. Catoni *et al.* (2013) recently provided evidence that temperature, and monthly rainfall to a lesser extent, could explain the seasonal variation of *R*_d_ in several Mediterranean maquis species. Galmés *et al.* (2007) noted that the number of studies on plant respiration responses to drought is generally limited, but particularly so for Mediterranean species. This is surprising considering the obvious importance of water stress in the Mediterranean region. Seasonal acclimation of respiration is believed to be more important in sclerophyllic perennial leaves (Galmés *et al.*, 2007; Zaragoza-Castells *et al.*, 2007, 2008) than in plants with short-lived leaves (review by Atkin and Macherel, 2009). A better characterization of the respiratory responses to drought relative to carbon gain is vital for elucidating the overall effects on carbon exchange dynamics in water-limited environments. Rainfall-exclusion experiments in natural ecosystems are laborious and expensive but are highly valuable to simulate more realistically long-term drought. Some studies have addressed the photosynthetic limitations under long-term drought in natural ecosystems comprising stomatal, mesophyll, and biochemical components (Limousin *et al.*, 2010; Martin-StPaul *et al.*, 2012). To the best of our knowledge, the effects of long-term experimental drought on photosynthesis in parallel with night and day respiration has not been investigated so far on mature species in natural ecosystems.

*Quercus ilex* L. is one of the ‘flagship’ species for the Mediterranean Basin because it is a typical evergreen sclerophyllic tree extending over a large geographical range and forms the terminal point of secondary succession over vast areas in the Iberian Peninsula, including low and higher altitudes, and near-coastal sites with an oceanic climate, as well as inland sites with a semi-arid climate (Lookingbill and Zavala, 2000; Niinemets, 2015). However, reduced stem growth and higher mortality rates found for *Q. ilex* in response to drought (Barbeta *et al.*, 2013) could decrease the distribution under predicted future drier conditions. Hence, *Q. ilex* is the ideal candidate to evaluate the seasonal acclimation of the foliar carbon balance in the long-term drought experiment of Prades (north-eastern Spain) where partial rainfall exclusion has been applied for the last 14 years, reducing soil moisture by an average of 13% (Ogaya *et al.*, 2014; Barbeta *et al.*, 2015).

We investigated the variations of foliar respiratory and photosynthetic traits of *Q. ilex* affected by seasonal changes in growth temperature and precipitation from winter to spring and summer. Furthermore, we studied the impact of long-term experimental drought on key limitations of photosynthesis comprising stomatal, mesophyllic, and biochemical components, as well as mitochondrial respiration during the day and night. Based on these parameters, we evaluated the response pattern of the foliar intrinsic water- and carbon-use efficiency (*WUE*_i_ and *CUE*_i_, respectively) with respect to the simulated drought. Our aim was to improve our understanding of the boundaries and mechanisms of foliar respiration and photosynthesis in terms of seasonal acclimation and adaptation to drought. We provide here a set of needed parameters that potentially help to improve model simulation of ecosystem carbon fluxes.

## Material and methods

### Experimental site

The experimental site was situated in the Prades Mountains in southern Catalonia (north-eastern Spain; 41°21′N, 1°2′E) at 950 m above sea level on a 25% south-facing slope. Temperature, photosynthetically active radiation, air humidity, and precipitation have been monitored continuously with a meteorological station installed at the site. The climate is Mediterranean, with a mean annual rainfall of 609mm and a mean annual temperature of 12.2 °C (climate data from the meteorological station for 1999–2012). The soil is a Dystric Cambisol over Paleozoic schist with a depth of 35–90cm. The forest is characterized by a dense, multi-stemmed crown dominated by *Q. ilex* and *Phillyrea latifolia* L. with a maximum height of 6–10 m. The understorey is composed of *Arbutus unedo* L., *Erica arborea* L., *Juniperus oxycedrus* L., and *Cistus albidus* L. A long-term rainfall-exclusion experiment has been established and maintained in this forest since 1999 to simulate *in situ* projected decreases in precipitation in the Mediterranean region (Peñuelas *et al.*, 2007). Four control and four treatment plots of 15×10 m were installed at the same altitude along the mountain slope. In the treatment plots, rain was partially excluded by PVC strips suspended 0.5–0.8 m above the soil (covering 30% of the soil surface). A ditch of 0.8 m in depth around the plots intercepted the runoff water from above the plots and conducted the water around to the bottom. The control plots received no treatment.

### Sampling method

**Fig. 1.**
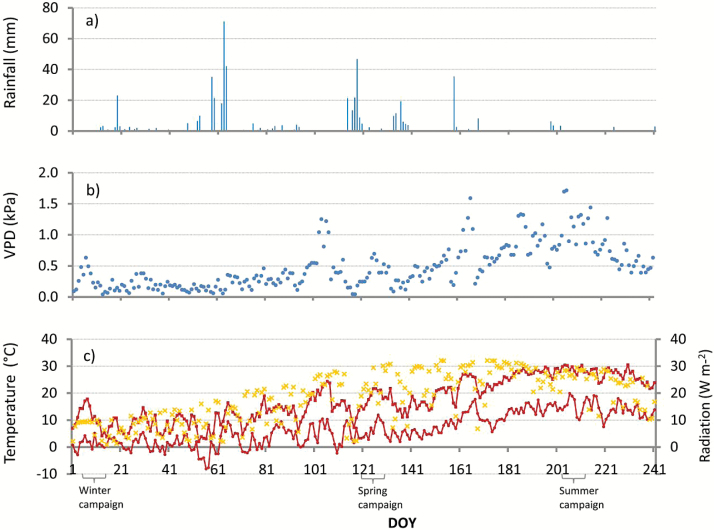
Environmental variables for the days of the year (DOY) from January to August 2013: rainfall (a), atmospheric vapour-pressure deficit (VPD) (b), and maximum and minimum temperatures (°C) (c) on the primary *y*-axes (red circles) and radiation (yellow crosses) on the secondary *y*-axes. The field campaigns are indicated.

We conducted three seasonal field campaigns: winter (5–11 January 2013), spring (30 April–4 May 2013) and summer (24–29 July 2013) (Fig. 1). Eight twigs for each drought and control plot (two replicates for each plot) were cut with a pruning pull from the sun-exposed crowns of *Q. ilex* trees. We recut the twigs under water in the field, wrapped them in plastic bags to minimize transpiration, and transported them in water buckets to a nearby laboratory. The twigs were pre-conditioned overnight in the laboratory at room temperature (22–26 °C), freshly recut in the morning, and then kept in dim light [50–100 photosynthetic photon-flux density (PPFD) in µmol photons m^−2^ s^−1^]. Once the leaves showed open stomata [stomatal limitation (*g*_s_)>0.03mol H_2_O m^−2^ s^−1^] and a stable stomatal internal CO_2_ concentration (*C*_i_, µmol CO_2_ mol air^−1^), we started the response curves. In a few cases, the twigs were kept for one or two additional nights until gas exchange was sufficiently stable to conduct a light-response curve. We have adopted this method to overcome limitations that we often faced in the field such as: (i) accessibility of the branches of mature trees (canopy height between 6 and 10 m); (ii) limited ability of the instruments to reach the standard leaf temperature (*T*_leaf_) of 25 °C; and (iii) unpredictable plant responses such as closed stomata or patchy stomatal conductance (Mott and Buckley, 1998, 2000). With the pre-conditioned twigs, in contrast, we reached stable gas-exchange values that are required for conducting a noise-free light- or CO_2_-response curve and to estimate reliably the photosynthetic potentials (see Supplementary Fig. S1 at *JXB* online). The leaves remained fresh and functional for several days controlled by stomatal conductance and fluorescent signals. The cut twigs showed stable values of night respiration for several days (see Supplementary Fig. S2 at *JXB* online). This method works well on Mediterranean oak species including *Q. ilex* as shown in other studies (Haldimann and Feller, 2004; Niinemets *et al.*, 2005; Sperlich *et al.*, 2015). This method provided us with the opportunity to look at the *potential* physiological properties under standardized conditions that are representative for each season of a control group and a drought treatment and that are independent of short-term meteorological variability (e.g. cloud cover, extreme temperatures, chilly periods, rain events) and unpredictable plant responses.

### Analyses of gas exchange and chlorophyll fluorescence

Gas exchange and chlorophyll fluorescence were measured with a Li-Cor LI-6400XT Portable Photosynthesis System equipped with a LI-6400-40 Leaf Chamber Fluorometer (Li-Cor, Lincoln, USA). Response curves of net assimilation versus PPFD were recorded in parallel with chlorophyll fluorescence measurements on mature, fully expanded leaves. In the summer campaign, we additionally conducted response curves of net assimilation versus CO_2_. Some of the *Q. ilex* leaves were too small to fill the leaf cuvette (2cm^2^), so the measured parameters were adjusted after the measurements. The leaves were prepared and acclimated prior to recording the response curves as described by Sperlich *et al.* (2014). First, we measured the maximum quantum yield of photosystem II (PSII; unitless) [*F*_v_/*F*_m_=(*F*_m_–*F*_o_)/*F*_m_] of a dark-adapted leaf (>30min) at ambient CO_2_ (*C*_a_ of 400 µmol CO_2_ m^−2^ s^−1^) and *T*_leaf_ of 25 °C. *F*_o_ is the minimal fluorescence measured under darkness, and *F*_m_ is the maximal fluorescence measured after a saturating light pulse. *F*_v_/*F*_m_ describes the fraction of absorbed photons used in photochemistry under dark conditions and serves as the primary stress indicator of the photosystems. Typical values range between 0.74 and 0.85. Ratios of <0.80 are indicative of induced photoprotection (sustained energy dissipation), and ratios <0.74 are indicative of chronic photoinhibition (Björkman and Demmig, 1987; Maxwell and Johnson, 2000; Verhoeven, 2014). We then acclimated the leaf to saturating light conditions (PPFD of 1200 µmol photons m^−2^ s^−1^) and simultaneously recorded gas exchange and chlorophyll fluorescence parameters at ambient CO_2_ and *T*_leaf_ as above: net assimilation rate (*A*_net_, µmol CO_2_ m^−2^ s^−1^), *g*_s_, *C*_i_, non-photochemical quenching (unitless) [NPQ=(*F*_m_ – *F*_m_’)/*F*_m_’, where *F*_m_’ is the maximal fluorescence of a light-adapted leaf], and the effective quantum yield of PSII (unitless) [Φ_PSII_=(*F*_m_’ – *F*_s_)/*F*_m_’, where *F*_s_ is the steady-state fluorescence of a light-adapted leaf].

We used the relationship of *A*_net_ versus *g*_s_ to estimate the foliar water-use efficiency (*WUE*_i_) which is defined as the amount of carbon gained per unit water used (Flexas *et al.*, 2013).The electron-transport rate based on the effective quantum yield of PSII (*J*_CF_ in µmol electron m^−2^ s^−1^) was calculated as

$$J_{CF}=\varepsilon \text{*}\Phi \text{PSII*}\alpha_{\text{L}}\text{*PPFD}$$ (1)

where ε is a scaling factor accounting for the partitioning of intercepted light between PSI and PSII. We assumed that light was equally distributed between the two photosystems (ε=0.5) (Bernacchi *et al.*, 2002; Niinemets *et al.*, 2005). The foliar absorbance (α_L_, unitless) was 0.932 for *Q. ilex* (Sperlich *et al.*, 2014). *J*_CF_ at ambient CO_2_ and saturating light was termed *J*_amb_.

### Light experiments

Light-response curves (*A*/PPFD) were generated by automatically applying changes in the photosynthetically active radiation with the LI-6400XT light source at a leaf chamber internal concentration (*C*_a_) of 400 µmol CO_2_ mol air^−1^. To obtain precise responses at the low range of the light gradient for estimating the daily mitochondrial respiration by the Kok effect (Kok, 1948), we used the following PPFD sequence (in µmol photons m^−2^ s^−1^): 2500→2000→1500→1000→800→600→500→400→300→200→150→125→100→75→50→40→30→20→10→5→0. The minimum and maximum times between each light level for the generation of the *A*/PPFD curves were set to 1 and 2min, respectively. The rapid changes in light levels prevented the correct adjustment of *T*_leaf_. We fixed the Peltier-block temperature (*T*_block_) in the leaf cuvette, so that *T*_leaf_ was 25 °C at the beginning of the *A*/PPFD curve. In the lower light levels where day respiration was estimated, *T*_leaf_ had dropped on average by 0.8 °C (standard error ±0.0004). Day respiration (*R*_d_ in µmol CO_2_ m^−2^ s^−1^) was estimated from the light-response curves with the method proposed by Yin *et al.* (2009) combining measurements of gas exchange and chlorophyll fluorescence. This method amended the Kok method (Kok, 1948) by substituting the *A*/PPFD relationship with *A*/(PPFD×Φ_PSII_/4) (see Yin *et al.*, 2009, for details on the protocol). We estimated night respiration (*R*_n_ in *µ*mol CO_2_ m^−2^ s^−1^) after darkening the leaf for 20–30min, ensuring that all reaction centres had been closed (controlled with *F*_o_). *R*_d_ and *R*_n_ were then normalized to unity at 25 °C with an Arrhenius function [parameter=exp(c – Δ*H*_a_/R*T*_k_)]. *T*_k_ is the leaf temperature (in Kelvin) and R is the molar gas constant (0.008314 kJ K^−1^ mol^−1^). The values of the scaling constant c (18.72, dimensionless) and energy of activation Δ*H*_a_ (46.39 kJ mol^−1^) for leaf respiration were taken from Bernacchi *et al.* (2001).

Thereafter, we applied a correction of *R*_d_ to account for the increase of *C*_i_ with decreases of PPFD described by Kirschbaum and Farquhar (1987). In this method, the intercept of plots of photosynthetic electron transport to PPFD is minimized through the adjustment of *R*_d_ via iteration (see Weerasinghe *et al.*, 2014, for details on the protocol).

With *R*_d_ from the light-response curves, we calculated the intrinsic carbon-use efficiency (*CUE*_i_) as proportion of carbon assimilated per carbon respired (Gifford, 2003), which served as a rough indicator for the foliar carbon balance (Pattison *et al.*, 1998; Galmés *et al.*, 2007):

$$CUE_{i}=1-\frac{R_{d}}{A_{net}}$$ (2)

### CO_2_ experiments

The *C*_a_ concentrations used to generate the CO_2_-response curves were 400→300→200→150→100→50→400→400→600→ 800→1200→2000 µmol CO_2_ mol air^−1^. *T*_leaf_ was set to 25 °C. The saturating PPFD used was 1200 µmol photons m^−2^ s^−1^ based on light-response curves conducted prior to the measurements campaigns. The results of all light-response curves after the measurement campaign, however, indicated a saturating PPFD of 1500 µmol photons m^−2^ s^−1^. The minimum and maximum times for stabilizing *A*_net_, *g*_s_, and *C*_i_ for each log were set to 4 and 6min, respectively. Diffusion leakage was corrected as described by Flexas *et al.* (2007).

### Estimation of mesophyll conductance

We estimated *g*_m_ (mol m^−2^ s^−1^ bar^−1^) using the variable-*J* method of Harley *et al.* (1992):

$$g_{m}=\frac{A_{\text{net}}}{C_{\text{i}}-\frac{\Gamma^{*}\left[J_{\text{CF}}+8\left(A_{\text{net}}+R_{\text{d}}\right)\right]}{J_{\text{CF}}-4\left(A_{\text{net}}+R_{\text{d}}\right)}}$$ (3)

where Γ* is the CO_2_ concentration at which the photorespiratory efflux of CO_2_ equals the rate of photosynthetic CO_2_ (37.43 ppm at 25 °C). Γ* and its temperature response were taken from Bernacchi *et al.* (2002). The chloroplastic CO_2_ concentration (*C*_c_ in µmol CO_2_ mol air^−1^) was determined as:

$$C_{c}=C_{i}-\frac{A_{net}}{g_{m}}$$ (4)

### Photosynthesis model

The photosynthesis model of Farquhar *et al.* (1980) considers photosynthesis as the minimum of the potential rates of Rubisco activity (*A*_c_) and ribulose-1,5-bisphosphate (RuBP) regeneration (*A*_j_). A third limitation (*A*_p_) was implemented that considers the limitation by triose-phosphate use at high CO_2_ concentrations when the CO_2_ response shows a plateau or decrease (Sharkey, 1985). The model was further modified by replacing *C*_i_ with *C*_c_ for the chloroplast where the actual carboxylation takes place (reviewed by Flexas *et al.*, 2008). As outlined above, we used the variable-*J* method for the *C*_c_ calculation to create *A*/*C*_c_ curves. The modelled assimilation rate *A*_mod_ was then calculated by the minimum of these three potential rates from the *A*/*C*_c_ curves:

$$A_{mod}=min\left\{A_{c},A_{j},A_{p}\right\}$$ (5)

where:

$$A_{c}=V_{cmax}*\left[\frac{C_{c}-\Gamma^{*}}{C_{c}+K_{c}\left(1+\frac{O}{K_{o}}\right)}\right]-R_{d}$$ (6)

where *V*_c,max_ (µmol CO_2_ m^−2^ s^−1^) is the maximum rate of Rubisco carboxylation, *K*_c_ is the Michaelis–Menten constant of Rubisco for CO_2_, *O* is the partial pressure of O_2_ at Rubisco, and *K*_o_ is the Michaelis–Menten constant of Rubisco for O_2_, taken from Bernacchi *et al.* (2002). The equation representing photosynthesis limited by RuBP regeneration is:

$$A_{j}=J_{1200}*\left[\frac{C_{c}-\Gamma^{*}}{4C_{c}+8\Gamma^{*}}\right]-R_{d}$$ (7)

where *J*_1200_ (in µmol electron m^−2^ s^−1^) is the rate of electron transport at a PPFD of 1200 µmol photons m^−2^ s^−1^ and saturating CO_2_. We assumed that *J*_1200_ became *J*_max_ under light and CO_2_ saturation when the maximum possible rate of electron transport was theoretically achieved, although we may have underestimated the true *J*_max_ (Buckley and Diaz-Espejo, 2015). The limitation of triose-phosphate use was estimated as:

$$A_{p}=\frac{3TPU*C_{c}}{C_{c}-\left(1+3\alpha_{TPU}\right)*\Gamma *}-R_{d}$$ (8)

where *TPU* is the rate of triose-phosphate use at saturating CO_2_ concentrations, and α_*TPU*_ is the proportion of glycerate not returned to the chloroplasts. Eqn 8 is from von Caemmerer (2000) after correcting a typographical error in the expression 3α_*TPU*_/2 to 3α_*TPU*_, as described by Gu *et al.* (2010). This equation fits the *A*/*C*_c_ curve plateau at high CO_2_ when a further increase in *C*_c_ does not produce any increase of *A*_net_ anymore or, in some cases, even produces a decline of *A*_net_.

In addition to the *A*/*C*_c_ curves, we replaced *C*_c_ with *C*_i_ in Eqns 6–8 and thus applied the above photosynthesis model to the traditional *A*/*C*_i_ curve. We used an adequate set of kinetic constants from Bernacchi *et al.* (2001). We considered *V*_c,max_, *J*_1200_, and *TPU* from the *A*/*C*_c_ curve as the ‘true’ biochemical potential to drive photosynthesis whereas the parameters from the *A*/*C*_i_ curve were the ‘apparent’ photosynthetic potential.

### Statistical analyses

We estimated the true and apparent values of *V*_c,max_, *J*_1200_, and *TPU* from Eqns 6–8 with the SOLVER Excel tool. SOLVER iteratively changes the parameters to minimize the sum of squares of the deviation of observed *A*_net_ versus modelled *A*_mod_. We then performed further statistical analyses with R version 3.0.2 (http://www.r-project.org/). Differences in the parameters between control and drought plots were determined with Student’s *t*-test (*P*≤0.05). The normality of the data was tested with the Shapiro–Wilk test, and the data was normalized if not normally distributed. One-factorial ANOVA with season as the main factor was tested for seasonal differences in the parameters. Significant differences were determined at *P*≤0.05 with Tukey’s honestly significant difference test. Linear regression analyses were conducted to study the relationships among various leaf traits such as *A*_net_/*g*_s_, *A*_net_/*g*_m_, *J*_1200_/*V*_c,max_, *g*_m_/*g*_s_, and *R*_n_ */R*_d_. We tested for differences in regression slopes and intercepts with analyses of co-variance (ANCOVAs).

## Results

### Environmental conditions over the sampling period

Frost events were frequent in winter and snowfall was also observed. The maximum temperatures during the day were on an average 4.9 °C (Table 1). The spring was humid with a precipitation comparable to that in winter (246 and 269mm, respectively) and was relatively cold (average of 12 °C) with occasional night frosts (Fig. 1). Spring together with winter accounted for nearly 80% of the annual average precipitation. The summer, in contrast, was dry and warm (total precipitation of 21mm and average temperature of 20.3 °C), with a vapour- pressure deficit (VPD) nearly twice as high as in spring (0.83 kPa) (Table 1). The partial rainfall exclusion reduced the soil water content (SWC) by a total of 13% from the beginning of the experiment in 1999 until the end of our measurement campaign in 2013. For the period of our measurement campaign, the SWC was on average 14% lower in the partial rainfall-exclusion plots compared with the control plots (Table 1). This difference was highest in spring, with a 24% lower SWC in the drought plots compared with the control plots.

**Table 1.** Dates and days of the year (DOY) for each season in 2013 with mean temperature (T), total precipitation (Prec.), mean vapour-pressure deficit (VPD), mean radiation, and the percentage of the difference in the soil water content between the control and drought plots (ΔSWC)

Season	Date	DOY	T (°C)	Prec. (mm)	VPD (kPa)	Radiation (W m^−2^)	ΔSWC (%)
Winter	1 January–21 March 2013	1–79	4.9	269	0.20	9.1	5.3
Spring	22 March–21 June 2013	79–171	12.0	246	0.45	21.3	23.9
Summer	22 June–31 August 2013	172–242	20.3	21.8	0.83	25.0	7.7
Total	1 January–31 August 13	1–242	12.1	537	048	18.3	13.5

### Seasonal changes in photosynthetic parameters

**Fig. 2.**
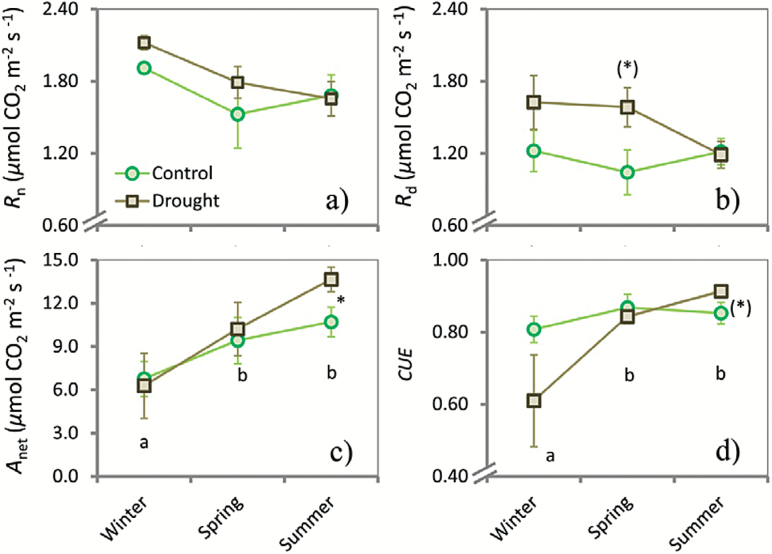
Line graphs depicting seasonal changes of night respiration (*R*_n_) (a), day respiration (*R*_d_) (b), net assimilation rate (*A*_net_) (c), and (d) carbon-use efficiency (*CUE*_i_) (d) for *Q. ilex*. Seasonal campaigns were conducted in winter, spring, and summer 2013. Asterisks and asterisks in brackets indicate significant (*P*<0.05) and marginally significant (*P*<0.1) differences between the control and drought plots for each season, respectively. Different lower-case letters indicate differences between seasons. Vertical bars indicate standard errors of the mean (*n*=59).

**Fig. 3.**
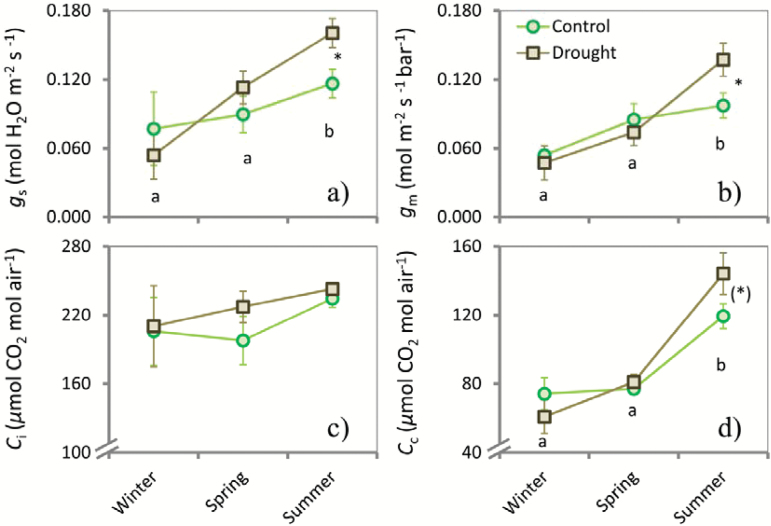
Line graphs depicting seasonal changes of stomatal conductance (*g*_s_) (a), mesophyll conductance (*g*_m_) (b), stomatal internal CO_2_ concentration (*C*_i_) (c), and chloroplastic CO_2_ concentration (*C*_c_) (d) in sunlit leaves of *Q. ilex.* Seasonal campaigns were conducted in winter, spring, and summer 2013. Asterisks and asterisks in brackets indicate significant (*P*<0.05) and marginally significant (*P*<0.1) differences between the control and drought plots for each season, respectively. Different lower-case letters indicate differences between seasons. Vertical bars indicate standard errors of the means (*n*=59).

**Fig. 4.**
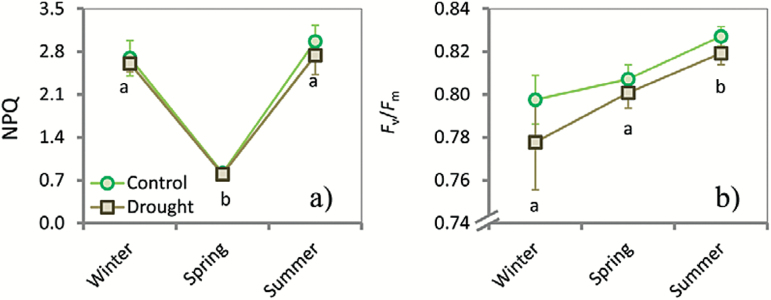
Line graphs depicting seasonal changes of non-photochemical quenching (NPQ) (a) and maximum quantum efficiency of PSII (*F*_v_/*F*_m_) (b) for *Q. ilex.* Seasonal campaigns were conducted in winter, spring, and summer 2013. Different lower-case letters indicate differences between seasons. Vertical bars indicate standard errors of the means (*n*=59).

We analysed the seasonality of the photosynthetic parameters using the full dataset independent of treatment. Winter had a strong effect on several parameters with lower average values than in spring and summer, except for *R*_n_ and *C*_i_ (Table 2). *A*_net_, *g*_s_, *g*_m_, and *F*_v_/*F*_m_ were significantly (*P*<0.05) and *R*_d_, *C*_c_, *CUE*_i_ were marginally significantly (*P*<0.10) lower in winter than in either spring or summer (Figs 2 and 3). In summer, we found the highest mean values of *A*_net_, *g*_s_, *g*_m_, and *C*_c_, which were significantly different from those in spring and winter (Fig. 3). *F*_v_/*F*_m_ was also highest in summer, demonstrating that the photosynthetic systems in spring had not yet fully recovered from the low winter temperatures but operated at peak efficiency in summer (Fig. 4b). NPQ is an indicator for photoinhibitory stress and dissipation of excess energy and was lowest in spring (significantly different from both winter and summer) (Fig. 4a). Neither Φ_CO2_ nor Φ_PSII_ differed significantly between the seasons (Table 2 and Supplementary Fig. S3 at *JXB* online). The saturating PPFDs for *A*_net_ and *J*_cf_ were 1484 and 1552, respectively, and did not change seasonally.

**Table 2.** Means (±standard errors) of a set of photosynthetic parameters and foliar traits for Q. ilex for the control group and the drought treatment in three seasonal campaigns (n=5–9)

Variable	Control			Drought		
	Winter	Spring	Summer	Winter	Spring	Summer
*R*_n_	1.91±0.03	1.53±0.28	1.68±0.17	2.12±0.06	1.79±0.13	1.65±0.14
*R*_d_	1.22±0.17	1.04±0.19	1.21±0.11	1.62±0.22	1.58±0.16	1.19±0.11
*R*_d_/*R*_n_	0.64±0.09	0.69±0.03	0.74±0.05	0.77±0.11	0.88±0.03	0.73±0.06
*A*_net_	6.76±1.2	9.43±1.0	10.71±1.0	5.52±2.0	10.17±0.7	13.66±0.9
*g*_s_	0.077±0.032	0.090±0.016	0.116±0.012	0.054±0.021	0.113±0.009	0.161±0.013
*g*_m_	0.054±0.009	0.085±0.014	0.097±0.011	0.047±0.017	0.074±0.017	0.137±0.014
*C*_i_	206±30	198±21	234±8	210±20	227±8	243±6
*C*_c_	74±9	77±3	119±7	61±10	81±5	139±23
NPQ	2.70±0.29	0.82±0.02	2.97±0.26	2.61±0.14	0.80±0.02	2.74±0.31
F_v_/F_m_	0.80±0.011	0.81±0.007	0.83±0.005	0.78±0.022	0.80±0.007	0.82±0.005
Φ_CO2_	0.0074±0.0020	0.0092±0.0009	0.0102±0.0014	0.0054±0.0014	0.0097±0.0008	0.0119±0.0018
Φ_PS2_	0.215±0.045	0.250±0.024	0.206±0.029	0.220±0.009	0.273±0.021	0.218±0.030
*V*_c,max_			107±9			120±11
*J*_max_			132±11			148±12
*TPU*			9.4±1.2			7.6±1.3

Several relationships were analysed with ANCOVAs to test whether seasonal changes in environmental conditions produced significant differences in slopes (Table 3). We analysed the relationship of *A*_net_/*g*_s_ as an indicator for *WUE*_i_. The slope of this relationship for the control group was significantly gentler in winter compared with spring and summer, indicating a lower *WUE*_i_. For the relationship of *A*_net_/*g*_m_, we analysed the effect of the mesophyll internal CO_2_ diffusion on net carbon assimilation. This relationship had a significantly steeper slope in winter in comparison with summer in the drought group. The relationship of *g*_m_/*g*_s_ unveils the relative contribution of stomatal and mesophyll diffusion limitation on the net carbon assimilation. The relationship of *g*_m_/*g*_s_ was significantly steeper in the control plot in spring in comparison with summer. We analysed the relative importance of day and night mitochondrial respiration with the relationship of *R*_d_/*R*_n_. The slope was significantly steeper in winter compared with spring and summer in both the control and drought plots.

**Table 3.** Regression equations and coefficients of determination (R^2^) for A_net_/g_s_, A_net_/g_m_, g_m_/g_s_, and R_d_/R_n_ for Q. ilex in three sampling campaigns in the control and drought plots *P* values indicate the significance of the differences between the slopes for the control and drought plots. Equations for non-significant relationships are not displayed (*n*=5–9).

Variable	Campaign	Plot	Equation	*R* ^2^	*P*
*A*_net_/*g*_s_	Total	Control	*y*=60.7*x*+3.68	0.72	0.417
		Drought	*y*=74.7*x*+1.92	0.88	
	Winter 2013	Control	*y*=36.1*x*+3.98	0.86	0.009
		Drought	*y*=94.9*x*+0.39	0.92	
	Spring 2013	Control	*y*=104.1*x*+1.51	0.98	0.380
		Drought	*y*=74.0*x*+2.71	0.68	
	Summer 2013	Control	*y*=79.1*x*+1.49	0.89	0.222
		Drought	*y*=53.9*x*+5.01	0.64	
*A*_net_/*g*_m_	Total	Control	*y*=79.3*x*+2.61	0.77	0.513
		Drought	*y*=70.2*x*+4.00	0.75	
	Winter 2013	Control			0.279
		Drought	*y*=115.1*x*+0.08	0.62	
	Spring 2013	Control	*y*=88.5*x*+1.01	0.92	0.521
		Drought	*y*=63.8*x*+5.17	0.80	
	Summer 2013	Control	*y*=88.8*x*+2.07	0.85	0.040
		Drought	*y*=30.5*x*+9.47	0.10	
*g*_m_/*g*_s_	Total	Control	*y*=0.254*x*+0.059	0.06	0.011
		Drought	*y*=0.757*x*+0.011	0.57	
	Winter 2013				–
		Drought	*y*=0.595*x*+0.017	0.56	
	Spring 2013	Control	*y*=1.051*x*+0.015	0.86	0.337
		Drought	*y*=0.637*x*+0.015	0.27	
	Summer 2013	Control	*y*=0.758*x*+0.009	0.75	0.949
		Drought	*y*=0.732*x*+0.020	0.30	
*R*_d_/*R*_n_	Total	Control	*y*=0.540*x*+0.263	0.59	0.0035
		Drought	*y*=0.980*x* 0.272	0.68	
	Winter 2013	Control	*y*=4.05*x* 6.14	0.78	0.279
		Drought	*y*=1.036*x* 0.343	0.61	
	Spring 2013	Control	*y* =0.639 *x*+0.063	0.96	0.0126
		Drought	*y*=1.147*x* 0.427	0.95	
	Summer 2013				–
		Drought	*y*=0.487*x*+0.373	0.38	

### Effect of experimental drought

**Fig. 5.**
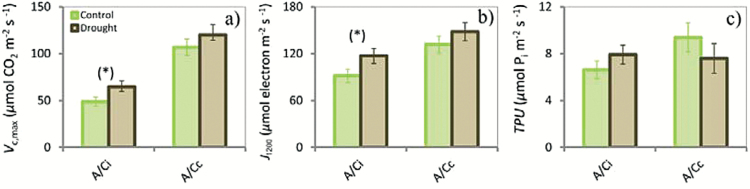
Bar graphs of maximum carboxylation rate (*V*_c,max_) (a), electron-transport rate at saturating light and CO_2_ (*J*_1200_) (b), and triose-phosphate use (*TPU*) (c) estimated with CO_2_-response curves based on *C*_i_ (*A*/*C*_c_) and *C*_c_ (*A*/*C*_c_) in the control and drought plots for the summer campaign. Marginal significant differences (*P*<0.1) between the control and drought plots are indicated by asterisks in brackets. Vertical bars indicate standard errors of the means (control, *n*=7; drought, *n*=8).

*R*_d_/*R*_n_ for all seasons combined was significantly higher in the drought treatment (0.79±0.06) compared with the control plots (0.71±0.03). No other general trends were detected. In the respective seasons, however, we found significant effects of the drought treatment, with several parameters showing higher average values compared with the control group (Figs 2 and 3): *A*_net_, *g*_s_, and *g*_m_ were significantly higher, and *CUE*_i_ and *C*_c_ were marginal significantly higher in summer, and *R*_d_ was marginally significantly higher in spring. We conducted carbon-response curves in summer only (see Material and methods). *J*_1200_, *V*_c,max_, and *TPU* were thus only available for the summer campaign. The drought treatment had no significant effect on these photosynthetic potentials when estimated from an *A*/*C*_c_ curve (Fig. 5). Additionally, we estimated the apparent photosynthetic potential from *A*/*C*_i_ curves. The drought treatment had a marginally significant effect on the apparent *J*_1200_ and apparent *V*_c,max_ with lower values in the control plot, but no effect on the apparent *TPU* (Fig. 5). A comparison of the photosynthetic potential from *A*/*C*_*i*_ and *A*/*C*_c_ curves indicated that the foliar internal diffusion limitation imposed by *g*_m_ accounted on average for a 54% higher *V*_c,max_ and a 30% higher *J*_1200_ and a 29% higher *TPU* with regard to the apparent photosynthetic potential.

**Fig. 6.**
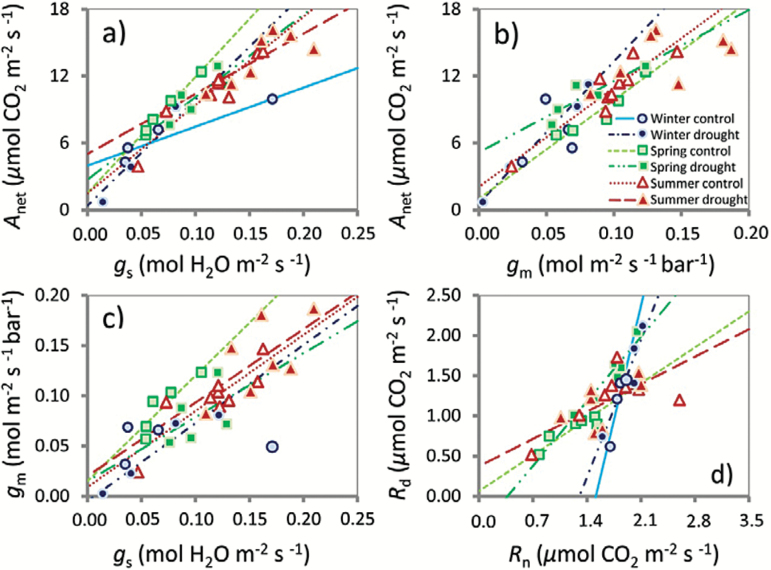
Scatter plots and regression lines of stomatal conductance (*g*_s_) versus net assimilation rate (*A*_net_) (a), mesophyll conductance (*g*_m_) versus *A*_net_ (b), *g*_s_ versus *g*_m_ (c) and night respiration (*R*_n_) versus day respiration (*R*_d_) (d) for each season and for control and drought plots. Only the regression lines for significant relationships (*P*<0.05) are displayed.

The ANCOVAs in the respective seasons identified significant differences in slopes as a result of the experimental drought. The slope of *A*_net_/*g*_s_ was significantly steeper in the control compared with the treatment group in the winter campaign, indicating a higher *WUE*_i_ in the control group (Fig. 6). The slope of *A*_net_/*g*_m_ was significantly steeper in the control group compared with the treatment group in the summer campaign (Fig. 6). The overall slope of *g*_m_/*g*_s_ was significantly steeper in the control group compared with the treatment group when all seasons were combined (Fig. 6). The slope of *R*_d_/*R*_n_ was significantly gentler in the control group compared with the treatment group in the spring campaign and when all seasons were combined. Neither season nor treatment significantly affected the slopes of *A*_net_/*R*_d_, *A*_net_/*R*_n_, *J*_amb_/ *A*_net_, and *C*_c_/*C*_i_ (Supplementary Tables S1–S4 at *JXB* online).

## Discussion

The scaling of carbon dynamics from one year to another is particularly challenging in Mediterranean environments due to climate extremes combined with a high interannual variability (Reynolds *et al.*, 1996; Morales *et al.*, 2005; Gulías *et al.*, 2009). We aimed to investigate the effect of seasonal changes in temperature and precipitation from winter to spring and summer on the photosynthetic and respiratory traits of a widely abundant Mediterranean tree species. However, abiotic stress under field conditions often hampers gas-exchange measurements due to deviations from the standard temperature (25 °C) or unpredictable plant responses, e.g. patchy stomatal conductance (Mott and Buckley, 1998, 2000), making it impossible to conduct response curves for the estimation of photosynthetic and respiratory parameters. Our data was thus obtained on cut twigs under standardized conditions in order to provide insights into the photosynthetic and respiratory potential independently of meteorological variability in the field. The cutting-twig method allowed us to analyse the *long-term acclimation* to the environmental conditions from which the twigs were derived and has been applied by other experts in the field (e.g. Epron and Dreyer, 1992; Niinemets *et al.*, 1999, 2005; Laisk *et al.*, 2002; Haldimann and Feller, 2004; Heskel *et al.*, 2014; Dusenge *et al.*, 2015). Our study thus provides a mechanistic description of seasonal changes in photosynthetic and respiratory processes under long-term drought that contributes to improve our understanding of the impacts of future climate change in Mediterranean-type ecosystems.

### Effect of seasonality on photosynthetic and respiratory traits

We found that cold winter temperatures had a stronger negative impact on the leaf physiology of *Q. ilex* than summer drought. The standardized *A*_net_ under controlled conditions (see Material and methods) was approximately half the rate in winter compared with the peak found in summer, yet relatively high average winter values were reached (6.5±1.3) that were comparable to those reported in other studies (Gratani, 1996; Ogaya and Peñuelas, 2003). Both *g*_m_ and *g*_s_ reduced the CO_2_ concentration in the chloroplasts in winter compared with spring and summer. In winter, however, *g*_m_ limited photosynthesis relatively more than *g*_s_. High water availability and low VPDs make the reduction of transpiratory water loss through stomatal closure less urgent in the winter period. There is some evidence that *g*_m_ acts as a stronger regulator for photosynthesis in winter (Sperlich *et al.*, 2014), although very few studies have examined the behaviour of *g*_m_ under natural winter conditions. Low temperatures in winter hamper photosynthetic metabolism and enzymatic activities (e.g. Corcuera *et al.*, 2004; Aranda *et al.*, 2005), which may account for a concurrent downregulation of photosynthesis through *g*_m_, as our results indicated. This was paralleled by a drastic decrease in the foliar carbon-use efficiency. In winter, chilly or freezing temperatures often coincide with clear skies and relatively high solar irradiances. The imbalance created between light energy absorbed in photochemistry and light energy used in metabolism increases the susceptibility to photoinhibitory stress (Demmig-Adams and Adams, 1992). This imbalance is particularly problematic for the evergreen vegetation, and thermal acclimation to winter conditions is essential to survive these adverse conditions (Blumler, 1991; Öquist and Huner, 2003). As a response, thylakoid membranes are reorganized, reaction centres are closed, and antennal size is reduced in order to protect the photosynthetic apparatus against overexcitation by the incoming radiation (García-Plazaola *et al.*, 1997; Huner *et al.*, 1998; Ensminger *et al.*, 2012; Verhoeven, 2014). The increased NPQ and decreased *F*_v_/*F*_m_ found in our study are good proxies for these photoprotective processes in the thylakoid membranes, indicating an increased thermal dissipation of excess energy and a decreased photochemical efficiency (Maxwell and Johnson, 2000). Thus, we found that *Q. ilex* acclimated to the winter conditions with reoccurring night frosts, and exploited the winter period photosynthetically at the cost of lower assimilation rates and a lower carbon-use efficiency (see also Hurry *et al.*, 2000; Dolman *et al.*, 2002; Sperlich *et al.*, 2014). We underline the fact that winter acclimation and exploitation can be essential for Mediterranean evergreen tree species to recover from stressful summer periods and to achieve a positive annual carbon balance.

Notably lower NPQs in spring indicate that the photosystems experienced the least amount of photochemical stress in this period. This is because the spring in our study was particularly cool and wet and was characterized by a low VPD. The high NPQ in winter and summer, in contrast, reflects strong photoprotection against photoinhibitory stress due to the temperature extremes. However, the photoprotective mechanisms seemed to be effective: the optimal light intensity for net assimilation and the electron transport (approximately 1500 µmol photons m^−2^ s^−1^ for both) and the effective quantum yield of PSII (Φ_PSII_) (Supplementary Fig. S3) did not change between the seasons.

The assimilation rates and the carbon-use efficiency increased from winter to spring, although it was not until summer that the peak photosynthetic activity was reached. The elevated *F*_v_/*F*_m_ underlines the fact that the photosynthetic apparatus fully recovered its maximum photochemical efficiency in summer. This contrasts with a very low total precipitation measured during the summer (22mm). However, *Q. ilex* can benefit from water reserves in deep soil layers and also in rock fractures (Barbeta *et al.*, 2015), which also explains its water-spending behaviour during drier periods (Sánchez-Costa *et al.*, 2015). It is known that *Q. ilex* develops a profound root system with a lignotuber that can make up as much as half of the total tree biomass (Canadell *et al.*, 1999), which is vital to withstand abiotic stress periods or disturbances. The precipitation in winter and spring together nearly reached the annual mean, so that deep soil water reserves are likely to have been yet filled in summer. High water availability in combination with high summer temperatures can account for the high photosynthetic activity in a potential water stress period. The replenishment of soil water reserves early in the growing season is critical to endure seasonal summer droughts in Mediterranean trees (Sperlich *et al.*, 2015). Pinto *et al.* (2014) also found the highest sap flow rates of *Quercus suber* L. in summer because its roots had access to the groundwater.

### Effect of rainfall exclusion on photosynthetic and respiratory traits

Drought experiments with rainfall exclusion under natural conditions can serve as valuable real-time model simulations for scenarios of future climate change. Unfortunately, long-term experiments over several years are costly and laborious and thus are particularly scarce. The rainfall exclusion in Prades, maintained since 1999, has reduced soil moisture by 13% with respect to ambient conditions and is probably the longest continuous-drought experiment worldwide (Wu *et al.*, 2011; Ogaya *et al.*, 2014).

Plants face a trade-off under water stress: the closure of the stomates reduces transpiratory water loss but at the same time constrains CO_2_ diffusion to the chloroplasts. Besides *g*_s_, *g*_m_ can act as a second leaf internal valve regulating the gas exchange through carbonic anhydrase and aquaporins and can thus help to prevent dehydration of vacuoles and cells (Terashima and Ono, 2002; Lopez *et al.*, 2013; Perez-Martin *et al.*, 2014). When chronic water stress begins to deplete stores of non-structural carbohydrates, plants are particularly reliant on photosynthetic products for refinement, repair, and protective actions (Niinemets *et al.*, 2009). We have provided novel evidence that *g*_m_ not only imposes an additional leaf internal resistance to gas exchange but can also *facilitate* the CO_2_ diffusion to the chloroplasts in comparison with a stronger control by stomata under drought. This was reflected by a comparatively higher *g*_m_ that increased the *g*_m_/*g*_s_ ratio under long-term drought (see also Galmés *et al.*, 2013). Our results are supported by recent findings obtained in *Q. ilex* and *Pinus halepensis* leaves showing a higher leaf internal CO_2_ conductance in parallel with a stricter stomatal control under severe drought (Sperlich *et al.*, 2015).

In addition to the importance of the diffusive capacity of stomata and mesophyll for the foliar carbon balance, this balance also depends strongly on the relationship of photosynthesis with respiration. However, the extent to which *R*_n_ or *R*_d_ are affected by water scarcity is highly uncertain. We found that *R*_d_ was approximately 74% of *R*_n_ and that the long-term rainfall-exclusion experiment increased the ratio of *R*_d_/*R*_n_ (0.79±0.04) compared with the control plot (0.71±0.03) due to a higher *R*_d_. Some studies found increased foliar respiration under severe water stress (Ghashghaie *et al.*, 2001), but reductions were also reported (Flexas *et al.*, 2006). The leaf may exert an acclimation of the respiratory metabolism through *R*_d_ because the demands for energy (ATP and NADPH) for synthesis of sucrose and carbon skeletons in the cytoplasm are higher under stressful conditions (Flexas *et al.*, 2006; Zaragoza-Castells *et al.*, 2007). *R*_d_ provides the basis for building up heat-stabilizing components such as heat-shock proteins or biogenic volatile organic compounds protecting the plant against detrimental effects (Tcherkez and Ribas-Carbó, 2012). Higher photoinhibitory stress can thus increase the respiratory metabolic activity expressed as a higher protein turnover at a given overall protein content (Niinemets, 2014; Weerasinghe *et al.*, 2014). This might explain the generally higher values of *R*_d_ that we found in the drought treatment. A lower stress level would evidently lead to a lower demand for energy and carbon skeletons and hence to a lower protein turnover. We found an indication for this in the effective photoprotective mechanism and lower rates of *R*_d_ in the summer campaign characterized by favourable conditions. In contrast to the results of Zaragoza-Castells *et al.* (2007), in our study only *R*_d_ but not *R*_n_ acclimated seasonally. The higher *R*_d_ in the drought treatment decreased significantly and coincided with the lower values of the control group, which remained unaffected throughout the seasons. This decrease of *R*_d_ in the drought treatment in summer—paralleled by higher rates of photosynthesis—significantly decreased *R*_d_/*R*_n_ and increased the foliar carbon-use efficiency. Griffin and Turnbull (2013) showed that a decreased *R*_d_/*R*_n_ can be explained by a suppressed light-saturated rate of oxygenation in photorespiration. Although we did not measure photorespiration directly, our data showed increased *g*_m_ and thus elevated CO_2_ concentrations in the chloroplasts (increase of 35% from spring to summer) (Fig. 3), which would benefit carboxylation over oxygenation. Overall, we found that *R*_d_—as the key player for the foliar carbon balance in *Q. ilex*—was the most responsive to seasons or treatment effects.

We found that the drought treatment had no significant effect on *J*_1200_, *V*_c,max_, or *TPU* in the summer campaign. Our results emphasize that the increased photosynthetic activity in the drought treatment in summer was not attributed to a higher potential in the biochemistry of photosynthesis, but rather than to an increased diffusive capacity of both *g*_s_ and *g*_m_. Interestingly, analysis of the apparent *J*_1200_ and apparent *V*_c,max_ (derived from *A*/*C*_i_ curves) identified marginally significant higher values in the rainfall-exclusion plot. This shows that the *A*/*C*_c_ method is more appropriate and that the traditional fitting method based on *A*/*C*_i_ curves may lead to confounding effects—as also shown in grapevines by Flexas *et al.* (2006). The foliar internal diffusion limitation imposed by *g*_m_ accounted on average for a 54% higher *V*_c,max_, a 30% higher *J*_max_, and a 29% higher *TPU* of the apparent photosynthetic potential. Similar values were reported for nearly 130 C_3_ species in a recent study by Sun *et al.* (2014).

We postulate firstly, that the summer period provided counterintuitively favourable conditions (as discussed above), and secondly, that the trees in the drought treatment acclimated most efficiently the balance between energy supply versus energy consumption in this period. Recent findings underscore the high plasticity of *Q. ilex* in response to seasonal changes in temperature or soil water compared with other Mediterranean species (Sperlich *et al.*, 2015). The rainfall-exclusion experiment in Prades was shown to result in a higher stem mortality (Barbeta *et al.*, 2013) and in a reduced leaf area in *Q. ilex* (Ogaya and Peñuelas, 2006), while increasing leaf mass per area (data not shown). With fewer leaves and lower total leaf area, the concentration of biochemical resources per leaf would increase. This might contribute to explaining the partly higher photosynthetic rates and carbon-use efficiency in the drought treatment. Sperlich *et al.* (2015) also found higher photosynthetic potentials in crowns that suffered a reduced total leaf area after a severe drought.

In this study, we examined the seasonality of photosynthetic and respiratory traits and evaluated the adaptive mechanism in response to reduced soil water under partial rainfall exclusion. A high climatic variability in the Mediterranean region can lead to counterintuitive effects, with the peak photosynthetic activity in summer, which is usually characterized by a high level of abiotic stress. The trees experiencing a 14-year-long drought treatment adapted through a higher plasticity in photosynthetic traits, so that eventually an atypical favourable growth period in summer was exploited more efficiently, with *g*_m_ and *R*_d_ as the determining parameters. Drought-induced growth declines may be attenuated in the long-term through morphological and physiological acclimation to drought (Leuzinger *et al.*, 2011; Barbeta *et al.*, 2013). Fewer leaves in the drought treatment were compensated by higher net photosynthetic rates. The similarity of photosynthetic potentials in the treatment and control plots suggests that there is also a dampening effect on the biochemical level.

## Supplementary data

Supplementary data are available at *JXB* online.

**Supplementary Fig. S1.** Four exemplary samples of carbon-response curves conducted at leaves of (i) twigs attached to the tree (field), (ii) after cutting and pre-conditioning the twigs under dim light in water in the lab for one night (day 1) and (iii) two nights (day 2).

**Supplementary Fig. S2.** Bar chart depicting the evolution of night respiration (*R*_n_) of *Q. ilex* directly after cutting the twig and after darkening for 30min (field), and at day 1, 2, and 3 after being pre-conditioned under dim light in water in the laboratory.

**Supplementary Fig S3.** Line graphs depicting seasonal changes of (a) quantum yield of CO_2_ (Φ_CO2_) and (b) effective quantum yield of PSII (Φ_PSII_) for *Q. ilex.*

**Supplementary Fig. S4.** Scatter plots and regression lines of maximum carboxylation rate (*V*_c,max_) versus maximum rate of electron transport (*J*_max_) derived from (a) *A*/*C*_c_ and (b) *A*/*C*_i_ response curves for control and drought plots in summer 2013.

**Supplementary Table S1.** Regression equations and coefficients of determination (*R* ^2^) for *A*_net_/*R*_d_ for *Q. ilex* in three sampling campaigns in control and drought plots.

**Supplementary Table S2.** Regression equations and coefficients of determination (*R* ^2^) for *A*_net_/*R*_n_ for *Q. ilex* in three sampling campaigns in control and drought plots.

**Supplementary Table S3.** Regression equations and coefficients of determination (*R* ^2^) for *J*_amb_/*A*_net_ for *Q. ilex* in three sampling campaigns in control and drought plots.

**Supplementary Table S4.** Regression equations and coefficients of determination (*R* ^2^) for *C*_c_/*C*_i_ for *Q. ilex* in three sampling campaigns in control and drought plots.

Supplementary Data
