# Supplementary material for: Balance between carbon gain and loss under long-term drought: impacts on foliar respiration and photosynthesis in Quercus ilex L
Source: J Exp Bot. 2015 Nov 9;67(3):821–33. doi: 10.1093/jxb/erv492 (PMC4737074; doi:10.1093/jxb/erv492)
Supplement: Supplementary Data [file supp_67_3_821__index.html]

Balance between carbon gain and loss under long-term drought: impacts on foliar respiration and photosynthesis in Quercus ilex L — Balance between carbon gain and loss under long-term drought: impacts on foliar respiration and photosynthesis in Quercus ilex L — Supplementary Data 

# Balance between carbon gain and loss under long-term drought: impacts on foliar respiration and photosynthesis in *Quercus ilex* L

## Supplementary Data

Data files

- supplementary\_figures\_S1\_S4\_tables\_S1\_S4.pdf - Supplementary Data
