# Supplementary material for: Balance between carbon gain and loss under long-term drought: impacts on foliar respiration and photosynthesis in Quercus ilex L
Source: J Exp Bot. 2015 Nov 9;67(3):821–33. doi: 10.1093/jxb/erv492 (PMC4737074; doi:10.1093/jxb/erv492)
Supplement: Supplementary Data [file supp_erv492_supplementary_figures_S1_S4_tables_S1_S4.pdf]

Journal of Experimental Botany

Balance between carbon gain and loss under long-term drought: impacts on foliar respiration and photosynthesis in *Quercus ilex* L.

Authors: D. Sperlich, A. Barbeta, R. Ogaya, S. Sabaté, J. Peñuelas

Supplementary Data

Supplementary Figures

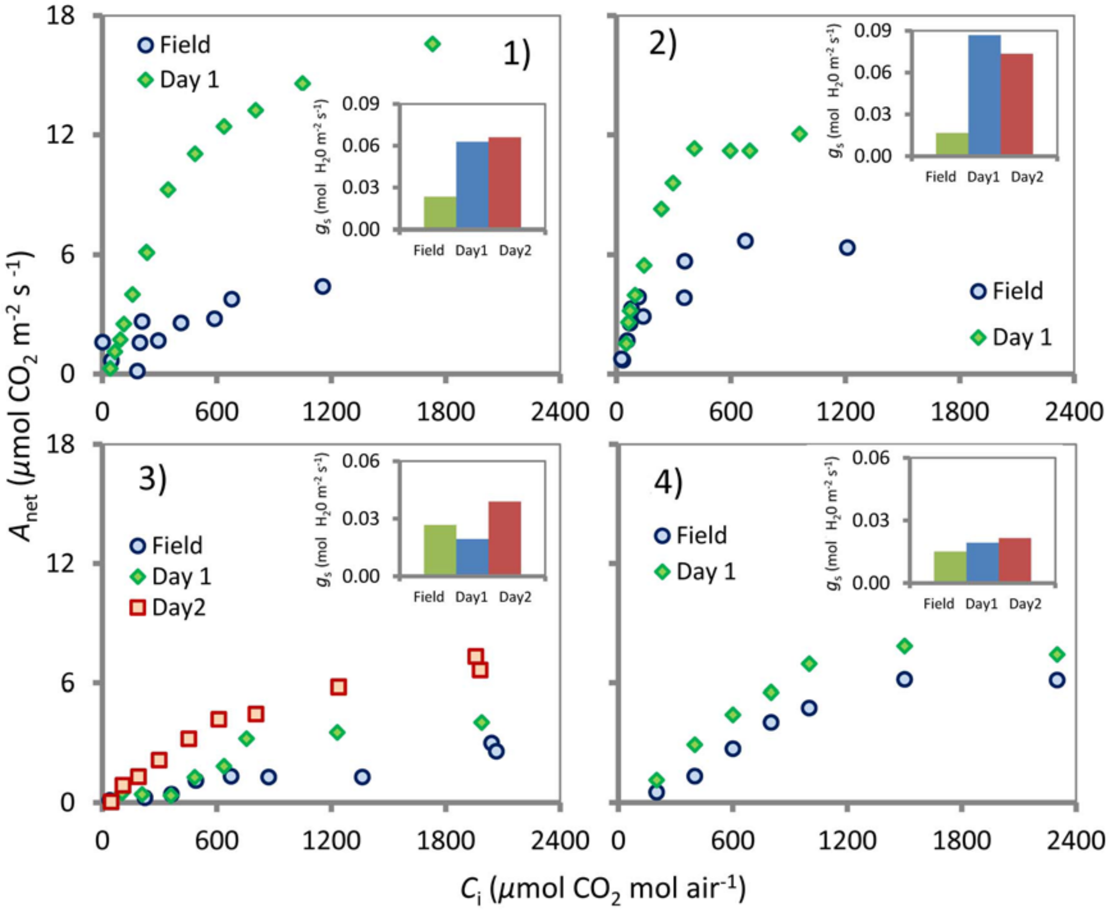

Supplementary Fig S1: Four exemplary samples of carbon-response curves conducted at leaves of i) twigs attached to the tree (Field), ii) after cutting and pre-conditioning the twigs under dim light in water in the lab for one night (Day 1) and iii) two nights (Day 2). Embedded bar charts show the evolution of stomatal conductance (g_s in mol H_2O m^{-2} s^{-1}) directly after cutting in the field, and after pre-conditioning at Day1 and Day2.

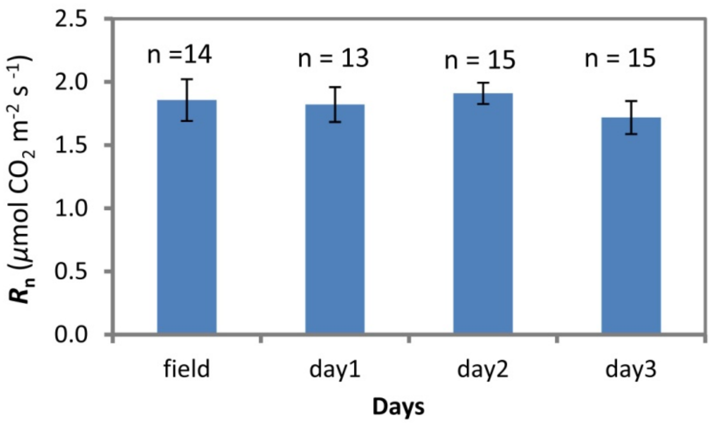

Supplementary Fig. S2: Bar chart depicting the evolution of night respiration (R_n) of a dark adapted leaf of *Q. ilex* after cutting in the field,ii) and at day one, two and three after being pre-conditioned under dim light in water in the lab. Vertical bars indicate standard errors of the means (n=13-15). No significant differences ($P < 0.05$) were found.

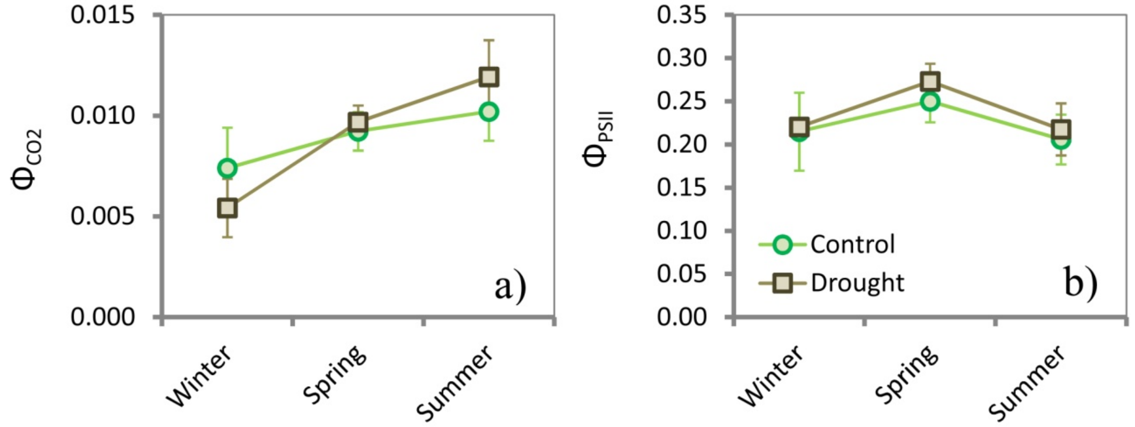

Supplementary Fig. S3: Line graphs depicting seasonal changes of a) quantum yield of CO_2 (Φ_{CO2}) and b) effective quantum yield of PSII (Φ_{PSII}) for *Q. ilex*. Seasonal campaigns were conducted in winter, spring, and summer 2013. Vertical bars indicate standard errors of the means (n = 59).

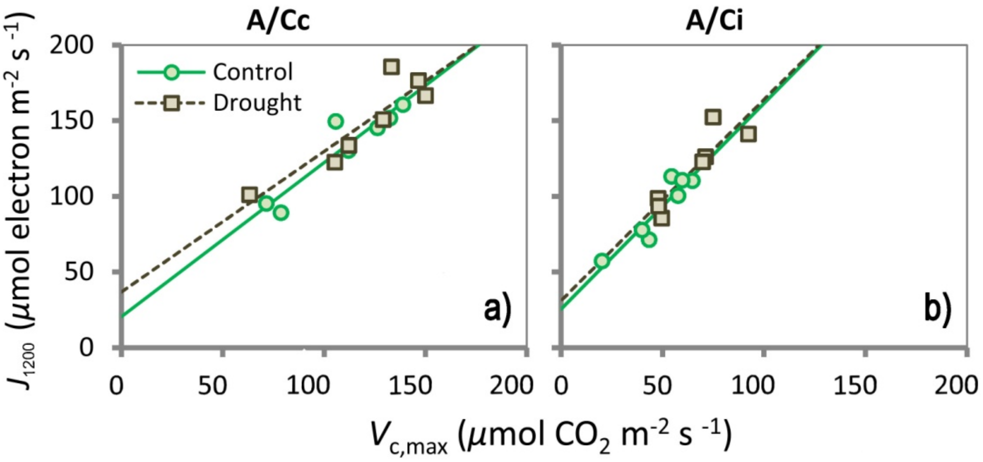

Supplementary Fig S4: Scatter plots and regression lines of maximum carboxylation rate ($V_{c,max}$) versus of electron-transport rate at saturating light and CO_2 (J_{1200}) derived from a) A/C_c and b) A/C_i response curves for control and drought plots in summer 2013. Only the regression lines for significant relationships ($P < 0.05$) are displayed.

Supplementary Tables

Supplementary Table S1. Regression equations and coefficients of determination (R^2) for A_{net}/R_d for *Q. ilex* in three sampling campaigns in the control and drought plots. The *P*-values indicate the significance of the differences between the slopes for the control and drought plots. Equations for non-significant relationships are not displayed.

Campaign	Plot	Equation	R^2	*P*
Total	control	y = -0.98x + 10.41	-0.04	0.513
	drought	y = 1.61x + 8.10	-0.03	
Winter 2013	control	y = 3.54x + 2.6	-0.04	0.400
	drought	y = 3.26x – 0.25	0.17	
Spring 2013	control	y = -2.37x + 11.10	-0.02	0.305
	drought	y = 0.89x + 8.4	-0.14	
Summer 2013	control	y = -2.81x + 14.18	-0.04	0.357
	drought	y = 6.61x + 6.02	0.65	

Supplementary Table S2. Regression equations and coefficients of determination (R^2) for A_{net}/R_n for *Q. ilex* in three sampling campaigns in control and drought plots. The *P*-values indicate the significance of the differences between the slopes for the control and drought plots. Equations for non-significant relationships are not displayed.

Campaign	Plot	Equation	R^2	*P*
Total	control	y = -1.46x + 11.69	0.02	0.129
	drought	y = 2.88x + 5.47	0.01	
Winter 2013	control	y = 22.2x – 33.4	0.45	0.427
	drought	y = 2.91x – 0.62	-0.13	
Spring 2013	control	y = -1.63x + 11.13	0.02	0.405
	drought	y = 1.43x + 7.39	0.02	
Summer 2013	control	y = -1.75x + 13.64	-0.04	0.205
	drought	y = 7.96x + 3.55	0.20	

Supplementary Table S3. Regression equations and coefficients of determination (R^2) for J_{amb}/A_{net} for *Q. ilex* in three sampling campaigns in control and drought plots. The *P*-values indicate the significance of the differences between the slopes for the control and drought plots. Equations for non-significant relationships are not displayed.

Campaign	Leaf position	Equation	R^2	*P*
Total	control	y = 4.64x + 87.2	0.29	0.711
	drought	y = 3.88x + 103.6	0.34	
Winter 2013	control	y = 6.61x + 76.5	0.27	0.738
	drought	y = 8.64x + 85.5	0.68	
Spring 2013	control	y =8.34 x + 63.8	0.59	0.538
	drought	y = 12.2x + 39.2	0.67	
Summer 2013	control	y = 4.61x + 78.8	0.16	0.543
	drought	y = 8.12x + 31.2	0.43	

Supplementary Table S4. Regression equations and coefficients of determination (R^2) for C_c/C_i for *Q. ilex* in three sampling campaigns in control and drought plots. The *P*-values indicate the significance of the differences between the slopes for the control and drought plots. Equations for non-significant relationships are not displayed.

Campaign	Leaf position	Equation	R^2	*P*
Total	control	y = 0.18x + 52.9	0.10	0.381
	drought	y = 0.011x + 81.8	-0.10	
Winter 2013	control	y = 0.171x + 39.0	-0.06	0.435
	drought	y = -0.02x + 57.2	-0.48	
Spring 2013	control	y =0.053 x + 63.8	-0.14	0.553
	drought	y = 0.12x + 48.3	0.74	
Summer 2013	control	y = 0.17x + 69.2	0.05	0.499
	drought	y = -1.09x + 344.4	-0.15	
